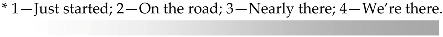# Propagating Change: Using RE-FRAME to Scale and Sustain A Community-Based Childhood Obesity Prevention Initiative

**DOI:** 10.3390/ijerph16050736

**Published:** 2019-03-01

**Authors:** Lynn Kennedy, Susan Pinkney, Selina Suleman, Louise C. Mâsse, Patti-Jean Naylor, Shazhan Amed

**Affiliations:** 1 British Columbia Children’s Hospital Research Institute, F503, 4480 Oak Street, Vancouver, BC V6H 3V4, Canada; Lynnkennedy93@gmail.com (L.K.); spinkney@bcchr.ca (S.P.); lmasse@bcchr.ubc.ca (L.C.M.); samed@cw.bc.ca (S.A.); 2 School of Population and Public Health, University of British Columbia, Vancouver, BC V6T 1Z3, Canada; 3 School of Exercise Science, Physical and Health Education, University of Victoria, Victoria, BC V8W 3P1, Canada; pjnaylor@uvic.ca; 4 Department of Pediatrics, University of British Columbia, Vancouver, BC V6H 3V4, Canada

**Keywords:** childhood obesity, health promotion, community-based participatory research, systems thinking, collective impact, knowledge exchange, capacity building, sustainability, scalability

## Abstract

Sustainable Childhood Obesity Prevention through Community Engagement (SCOPE) has developed Live 5-2-1-0, a multi-sectoral, multi-component community-based childhood obesity prevention initiative grounded in systems thinking and participatory research principles. Building on previous work, this study continued implementation of an innovative knowledge exchange model, RE-FRAME, in two ‘new’ and two ‘existing’ Live 5-2-1-0 communities. This mixed-methods study examined follow-up data to determine the nature and extent of the model’s ability to build and sustain community capacity and facilitate the scale-up and sustainability of systems- and community-level change. Qualitative and quantitative data were collected using stakeholder interviews, and quantitative process tracking (PTT) and capacity building tools (CCBT), and were analyzed using thematic analysis and descriptive statistics, respectively. Results from three communities with baseline and follow-up CCBT data showed capacity domain scores increased (15/27; 56%) or remained constant (10/27; 37%) over the study period. PTT data showed over 50 multi-sectoral community partnerships formed in Community D (new) and 108 actions implemented. Stakeholder interviews identified having a common cause, co-ownership, champion networks and consistency of the Live 5-2-1-0 message as essential to sustainability of the initiative. RE-FRAME supports knowledge exchange and community capacity-building that is integral to initiating and sustaining a community-based childhood obesity prevention initiative.

## 1. Introduction

One in three children in Canada are affected by childhood overweight or obesity [1], a complex, multidimensional problem that is linked to elevated risk of developing serious life-long conditions like type 2 diabetes and heart disease [2]. To address this complex problem, Sustainable Childhood Obesity Prevention through Community Engagement (SCOPE) has developed a multi-sectoral, multi-component childhood obesity prevention initiative called Live 5-2-1-0. Through this initiative, SCOPE partners with communities in the province of British Columbia (BC), Canada to engage a range of community stakeholders (e.g., in local government, health, education, businesses, etc.) to share a consistent, evidence-based [3,4] message (i.e., 5+ vegetables and fruits, <2 h of screen time, at least one hour of physical activity, and zero sugary drinks per day) and implement action to create and sustain healthy environments for children. This type of capacity-building approach grounded in systems thinking and principles of community-based participatory research and collective impact has proven to be an effective response to the complex socio-ecological causes of childhood obesity [5,6,7,8,9,10,11].

In 2009, SCOPE first partnered with two pilot communities to conceptualize this initiative in the BC context and has since progressed through several phases of implementation. Phase 1 (2009–2012) was a developmental phase of community engagement and co-creation that led to the identification of Live 5-2-1-0 as the message and common agenda [12] that would frame the initiative. Phase 2 (2013–2016) focused on community-led action with stakeholders from multiple community sectors implementing unique and/or adapted ideas and resources to share and support the message. Through this initial experiential learning, a novel knowledge exchange (KE) model called RE-FRAME emerged which describes the processes that occur within and between communities, as well as between SCOPE’s central team and partner communities, which facilitate effective and sustainable community-led systems-level change (Table 1). 

A protocol for implementing and evaluating the RE-FRAME model has been described in an earlier publication [13], including baseline data illustrating the model’s utility in supporting and sustaining multi-sector partnership growth and action implementation in two ‘existing’ (implementing for >2 years) Live 5-2-1-0 communities, and in initiating implementation in two ‘new’ (implementing for ≤1 year) partner communities. The primary objective of this study was to collect and examine follow-up data to determine whether the RE-FRAME KE model has:

(1) Enabled effective scale-up of implementation in ‘new’ Live 5-2-1-0 partner communities;
(2) Increased community capacity in new and existing communities to support and sustain Live 5-2-1-0 implementation.

A second objective was to describe how the KE mechanisms facilitated sustainable community action and increased community capacity.

## 2. Materials and Methods

### 2.1. The Knowledge Exchange Model—RE-FRAME

This model (Table 1), which has been described in detail in a previous publication [13], was designed to support communities to build their capacity to engage multisectoral partners to implement and sustain local action in support of Live 5-2-1-0. RE-FRAME aims to help existing partner communities to sustain implementation of Live 5-2-1-0 and also support new communities interested in implementing the initiative. Scale-up to new communities involves the provision of resources and linkages to existing knowledge and evidence-informed practices, while at the same time, supporting the adaptation of the initiative so that it meets unique community contexts.

**Figure 1:**
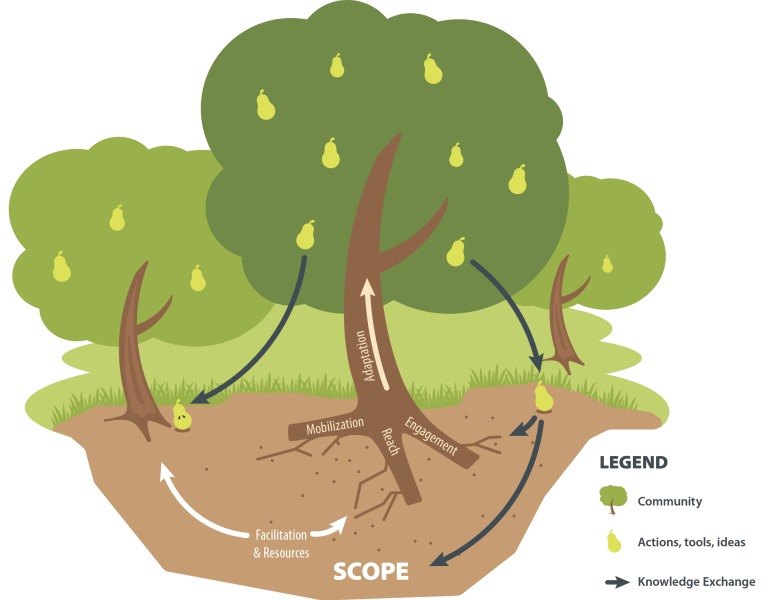
Implementing Live 5-2-1-0 via the RE-FRAME Model.

Figure 1 illustrates the operationalization of the RE-FRAME model. A local community (the tree) engages with SCOPE via a local ‘backbone’ organization (i.e., local government, Division of Family Practice) or a local committee that is comprised of a group of leaders who have identified child health and wellness as a priority. Engagement with SCOPE (the soil) allows a community to engage with a network (the forest) of Live 5-2-1-0 communities, and to access:

- Free, customizable resources (e.g., toolkits, posters, ‘how to’ guides, etc.) through the Live 5-2-1-0 Online Resource Map;
- Quarterly webinars, annual face-to-face summits, and training events to connect stakeholders within and between Live 5-2-1-0 communities to facilitate exchange of stakeholder experiential learning and adaptation of implementation processes, as well as provide coaching and training;
- Ongoing and ad hoc central support to various stakeholder groups for all aspects of Live 5-2-1-0 implementation and monitoring including: community engagement across multiple community sectors, coordination and alignment with regional and provincial initiatives, new resource development and/or adaptation of existing resources, implementation and evaluation of sector-specific projects (i.e., with private business owners or family physicians), analysis and reporting of community-specific process data, among others; and
- Via a two-way linking system (that has been previously described), linkages between the SCOPE team and community stakeholders so that ideas, solutions, tools, and best practice, as well as community priorities, contexts and strengths, can be shared [13].

These components that make up SCOPE’s ‘fertile soil’ support communities to reach, engage, and mobilize (via the roots of the tree) multi-sectoral stakeholder partners and have proven integral to successful community-led efforts for childhood obesity prevention [14,15]. The result is community-led actions, ideas, tools and initiatives (the fruit) that, when shared via SCOPE’s KE platform, contribute to increased capacity across a network of Live 5-2-1-0 communities (the forest) and also, plant a seed of interest in new communities, expanding the provincial Live 5-2-1-0 initiative even further [13].

### 2.2. Participating Communities

Four BC communities participated in the study: two ‘existing’ communities (A and B) that were heavily involved in the initial partnership with SCOPE (since 2009 and 2012, respectively) and contributed to the experiential learning that led to the creation of RE-FRAME, and two ‘new’ communities that began implementing the Live 5-2-1-0 initiative in 2014, and were less involved in the creation of the KT model. Communities A and B are both large cities that are located adjacent to each other (population 141,498 and 86,857 respectively), community C is a city that consists of multiple small municipalities (population 29,348), and community D is a small remote city (population 6746).

### 2.3. Data Collection and Analysis

Using a mixed-methods study design guided by Graham’s knowledge to action cycle [16] and the RE-AIM framework [17,18], we collected data to examine the nature and extent of the model’s ability to build community capacity and support communities in sharing or supporting the Live 5-2-1-0 message—i.e., translating knowledge to action. We employed a variety of qualitative and quantitative data collection tools including SCOPE’s Partnership Tracking Tool (PTT), the Public Health Agency of Canada’s (PHAC) Community Capacity Building Tool (CCBT), and semi-structured interviews with a local stakeholder from each participating community who was responsible for coordinating the initiative locally. CCBT was completed by a local stakeholder committee at baseline, with initial CCBT measurements completed from November 2014 to February 2015; follow-up CCBT measurements were completed by the same committee between February to April 2016. Baseline qualitative interviews were completed between November 2014 to January 2015, with follow-up interviews completed in January and February 2016. PTT data was collected throughout the study, with data collected for ‘existing’ communities from 2012–2016 and from 2014–2016 for ‘new’ communities (following their entry into the study).

The PTT and CCBT have been described in detail previously [13], although we provide a brief overview of each below:

- PTT: A proprietary online progress-tracking platform (username- and password-protected) that allows key community stakeholders to enter data about local partnership formation (e.g., partner organization, sector represented, stage of partnership) and resulting outcomes (e.g., actions implemented and Live 5-2-1-0 resources shared via the partnership).
- CCBT: A valid, reliable tool developed by PHAC that measures capacity in nine evidence-based community capacity domains (Table 2) using a Likert scale (1—Just started; 2—On the road; 3—Nearly there; 4—We’re there) [19]. A community’s capacity in each domain reflects the mean score received for the subset of related response items (2–4 per domain), where all scores are determined by community stakeholder consensus (e.g., at a monthly partnership meeting).

Quantitative data were analyzed using descriptive statistics (means, frequency, proportions). Semi-structured interviews with local community coordinators (who led Live 5-2-1-0 within their communities) were conducted as described previously [13]. All interviews were transcribed verbatim. Qualitative data from semi-structured interviews were then analyzed using iterative thematic analysis whereby diverse sections of data were grouped into smaller analytic codes [20]. Thematic analysis was followed by repeat analysis and grouping by multiple study team members (Lynn Kennedy, Susan Pinkney, Selina Suleman) to arrive at a final list of thematic codes. This was similarly achieved through repeat-analyses by study team members (Lynn Kennedy, Selina Suleman). 

## 3. Results

### 3.1. Multi-sectoral Partnerships Initiated and Sustained Multi-setting Action

Full PTT data for Communities A and B (existing) from 2012–2015 has been reported previously [13]. Community D (new) completed full data entry for the PTT, allowing multi-pronged analysis of the initiative’s growth during the first two years of Live 5-2-1-0 implementation. Community C (new) was unable to report on partnerships and resource distribution due to staff turnover and limited capacity for local data collection. The Live 5-2-1-0 actions implemented by Community Cover the first year of their participation in the study have been reported previously [13].

**Figure 2:**
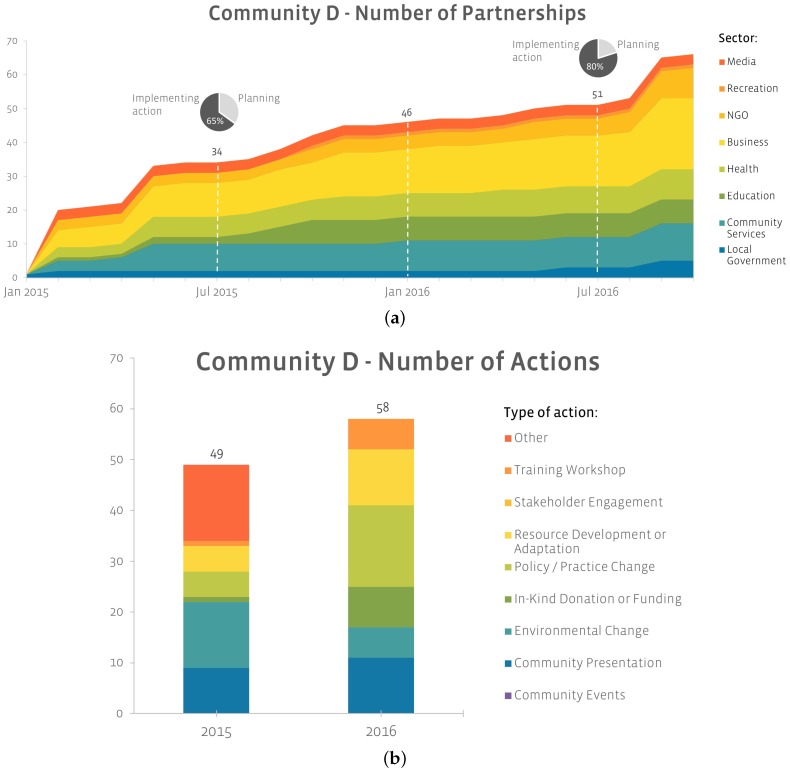
Live 5-2-1-0 Partnership Formation, Growth, and Action in ‘New’ Communities. (**a**) The formation and growth of multi-sectoral community partnerships in Community D (new) using the PTT. (**b**) Actions implemented by Community D (new) over the study period.

Figure 2a depicts the formation and growth of multi-sectoral community partnerships in Community D (new) where nearly 50 partnerships were formed in the first year of implementation. By June 2016, 80% of the 51 community partnerships had progressed from ‘planning action’ to ‘implementing action’. By the end of the study period, partners implementing Live 5-2-1-0 represented all 8 community sectors, with private businesses most heavily represented (32%), followed by community services (17%), the health sector (9%) and NGOs (9%).

Over this same time-period, partners in Community D implemented 108 actions (Figure 2b) and distributed over 2200 resources (e.g., fact sheets, school newsletters, the Healthy Habits Questionnaire, etc.) in support of Live 5-2-1-0. A modest growth of 16% was observed in the number of actions implemented from Year 1 (2015) to Year 2 (2016). Notably, Community D began implementing environmental and policy/practice changes in its first year of implementation. Examples of actions implemented by stakeholders in Community D include:

- The library stopped serving juice and animal crackers at their pre-school aged story time and began serving fruits and veggies instead;
- The front desk staff at the local Medical Clinic started handing out Healthy Habit Questionnaires to all children or their parents/guardians for discussion with a care provider during primary care visits;
- Students were taken on a supermarket tour to explore healthy food choices, according to the Canada Food Guide and linked to the Live 5-2-1-0 message.

Community C showed similar capacity to implement actions early in Live 5-2-1-0 implementation. In particular, nearly 60 actions were completed in the first full year of implementation, of which approximately 75% were community presentations or events.

### 3.2. Community Capacity

#### 3.2.1. Community Capacity Increased over Time

Three communities (one existing, two new) completed the CCBT at both baseline and follow-up. Follow-up CCBT data collection was not possible for Community A (existing) due to a suspension in meetings of the local stakeholder committee that completed the tool at baseline. Both new and existing communities demonstrated an increase in overall mean capacity domain score over a one-year period (Table 3).

The starting point for community capacity varied between existing versus new communities. In the one existing community with complete CCBT data (Community B), community capacity continued to develop and had reached an advanced state over the study period. Existing communities reported community capacity as being ‘nearly there’ at baseline and Community B reported capacity as “we’re there” 1-year later. In contrast, both newer communities reported they were partway between “on the road” and “nearly there” on the CCBT scale at baseline, and one year later reported they were at (Community D) or approaching (Community C) the “nearly there” mark.

Table 3 exhibits the changes observed from baseline to follow-up in each CCBT domain for Communities B, C, and D. In looking at the domain scores of all three communities with baseline and follow-up data together (three communities with nine CCBT domain scores each, for a total *N* = 27), most domain scores increased (15/27;56%) or held constant (10/27;37%) over the study period. For community B that was an existing Live 5-2-1-0 community, these data support sustained capacity building. Notably, perceived capacity across all three communities increased in three key domains: leadership, community structure, and role of external support. Further, while existing communities tended to report slightly higher capacity at baseline, there were domains where new communities were already proficient—and even ahead (i.e., role of external support, resource mobilization)—at baseline.

#### 3.2.2. What Increases Community Capacity?

Local coordinators from each participating community were interviewed twice over the study period (*N* = 8 interviews) to better understand what aspects of the Live 5-2-1-0 initiative and its implementation processes contributed to changes in community capacity and the sustainability of action over time. Key themes and subthemes emerged:

(i) A Common Agenda and Co-ownership Cultivates Partnerships

Communities highlighted the role of Live 5-2-1-0 in creating a common cause/goal/agenda as a starting point for communities to build partnerships and to engage and mobilize local champions. This was highlighted as being particularly critical in the early stages of implementation. Having a broad yet cohesive agenda of promoting and supporting healthy childhood behaviours using the common Live 5-2-1-0 message enabled diverse partners to conceive of how their individual organizational priorities and activities were connected to and could support the work, and also be supported by this work.

- “I think there just needs to be that shared vision and that shared belief in the message, that this is something that is important to each respective organization, and we think it’s important for the community, and that’s why we’re here.” (A1)
- “The [city’s] healthy communities committee had a strategic planning day…and decided on three priorities, one of which was childhood health and wellness. The committee decided that they would focus on the Live 5-2-1-0 messaging as a strategy to deal with childhood health and wellness.” (C1)

Related to this, the co-creation and co-ownership of local resources and projects that grew out of support for Live 5-2-1-0 was also identified as a unifying force and mechanism for deepening and maintaining partnerships. Co-creation is a process of collaborative development that brings key stakeholders together to jointly produce an outcome [21]. Co-creation is embedded in the implementation process of Live 5-2-1-0 such that production of new and adaptation of existing ideas and resources is flexible and communities are encouraged to lead the process locally, drawing on external expertise and support from SCOPE as needed. Particularly for existing communities where the initiative has been around longer, the process of co-creating and co-owning Live 5-2-1-0 resources and/or projects played a vital role in strengthening partnerships between sectors, maintaining buy-in after the initial enthusiasm for the message may have subsided, enhancing credibility of Live 5-2-10 resources and projects, and enhancing the relevancy of the initiative for partners.

- “I think there needs to be a collaborative project. There needs to be something that gives us a reason to come together and something that’s tangible that we can work towards.” (A1)

Local resources or projects that were identified by communities as having been effective in both building buy-in, cementing partnerships and ensuring ongoing support included the co-creation of a Live 5-2-1-0 Medicine Wheel resource in collaboration with local Indigenous partners, as well as the development of a Live 5-2-1-0 Service Providers’ Toolkit that involved extensive and continuous engagement and input from a broad range of local partners; this occurred in response to experience gained during development of an earlier toolkit:

- “…One learning we had from the Physician Toolkit, that we didn’t do enough physician engagement around developing the Toolkit. So, that can be improved. And that was big with regards to the Service Provider Toolkit—and through that there is more ownership on what is being created.” (B2)

Another locally-led and managed project that several communities cited as being powerful in building and sustaining collaborative partnerships were Live 5-2-1-0 Playboxes (sturdy metal bins installed in city parks and filled with equipment for outdoor activity, that are free to unlock and use with a shared code for a combination lock):

- “I think a big thing for us again are the Playboxes. So when we are talking about shared responsibility, which I think increases the sustainability—to have all those different partners in the community to partner on the Playboxes, with either financial or in-kind contribution, and then they have their logos on the Playboxes which has made it that much more financial sustainable… I think the more partners you have engaged and visible in the community promoting Live 5-2-1-0, specifically the Playboxes, the more sustainable it becomes, because no one wants to see it taken away and their names attached to it.” (B2)

(ii) Champion networks spark interest and fuel sustainability

Community champions are individuals within a community who voluntarily take on a role of passionate advocacy and leadership for an idea or project, and leverage their networks to expand support [22]. Champions catalyze the building and strengthening of local partnerships and are key to sustaining the Live 5-2-1-0 initiative within a community.

- “…they’re actively looking for those opportunities for the 5210 message to be further integrated and shared in our community…they’re the ‘ideas’ people… they’re actively looking for new places for this work to expand…” (A1, describing qualities of a champion)
- “I think the challenge is … just finding somebody who truly believes in the message so that you have a champion, because as it shows with the school district, if you have somebody that is a champion then you will get really far but if you don’t have that champion in place it just sort of stalls the whole progress.” (C1)

In addition to initial advocacy, a core group of local champions who could expand and manage relationships was often described as an important part of maintaining engagement. The importance of laying credible groundwork with partners was highlighted, as well as being able to connect the aims of the initiative to ongoing community activities. As networks were leveraged and built upon to engage additional partners and expand implementation, there were also challenges of capacity and expectations that were encountered. For example, it was recognized that building relationships took time, and that new ‘converts’ were empowered to take action through the provision of existing resources that could be used immediately, connections to activities that they were already doing, or involvement in collaborative shared projects as outlined above.

- “We have a couple new partners that have come on board, and I believe that the reason that those organizations have expressed an interest in becoming a partner is just we’ve had numerous face to face conversations, where we’re exchanging knowledge, where we’re building relationships, and I don’t think it can happen without that.” (A1)
- “…it has been very collaborative. It’s been pretty much across the sectors, with the media, business partners, or the health and wellness folks…And I think our approach on the way we went about it was good, starting with the inner circle to working its way out. I think that it gave the messaging some integrity and leveraging.” (D2)
- “… time is often the barrier but I think just trying to jump on board and create partnerships so that it’s really not a lot of additional work. Say for example, with the story time in the park, it really was no additional work because the story time in the park was already going on and so all I needed to do was give them the [Live 5-2-1-0] tips and they stuck them in the books that they were handing out so it wasn’t any additional work for partners and I think that’s one of the big things. Create these partnerships and just trying to climb on board with things that are already going on so that it’s not really that much additional work and then once you get those easy wins then looking at the harder stuff that maybe does take a bit more time and effort.” (C1)

(iii) Sustainability requires consistency, recognition and integration

Reflecting from a year or more into implementation of the initiative, communities highlighted the notion that visibility, consistency and entrenchment of the Live 5-2-1-0 message and initiative lends it credibility, opens the door to more partnerships and buy-in from various sectors, and facilitates uptake by stakeholders—which then feeds back into further expansion of visibility, perpetuating the cycle of uptake and support.

- “…getting into the consciousness, getting out there, spreading the messaging, and that just leads to more uptake as we go. People are hearing more of the message, they are getting excited about it, they want to share it with their students. And I think that is the strength of their message that is really valuable. Just spreading it out into the community helps with the sustainability. It is a good, simple, strong messaging. I think it’s this kind of stuff that leads to sustainability for sure.” (D2)
- “I think because the message has been in the community long enough, that people know that it’s here to stay, that is not some sort of an aside that the city is going through. So, it’s easier to get organizations on board with distributing materials or allowing us to come in and do presentations.” (A2)
- “I think again because the message is so well established, it’s really easy for people to say yes to it. They do not have to wonder if it’s a good message, is there going to be any blow-back from parents or the community if we start to support this.” (A2)

In addition, new communities noted that acknowledging and celebrating indicators of progress and moments of success played an important role in generating enthusiasm, buy-in and continued momentum within communities.

- “We keep on having those moments, where we see the success of it. We interviewed someone last week and they were really excited about how we had healthy food and beverage messaging. And we say we’re 5-2-1-0 for after school child care and they’re like ‘I think that’s so great you know’. So lots of these moments that make it really worthwhile for us I guess.” (C2)
- “For us, we have these awesome moments with it. … It’s just we kept having comments where our people were like ‘there’s no way kids will eat fruits or vegetables, there’s no way they’re going to have any interest in working in the community garden’. And it’s really funny to see from a year ago, where they didn’t think they would eat it and they didn’t think they would participate and now they’re like ‘they were so excited for that!’ and ‘they’ve been eating so many raspberries all summer!’ or whatever.” (C2)

Finally, this was seen as leading to integration of the Live 5-2-1-0 message and implementation of supportive action within partners’ practices, and the introduction of small policy changes that alter and improve the way things are done in sectors across the community.

- “I think for us the big thing is training with staff, so any new staff hired, it’s part of their orientation. It’s in the rec department, to be honest, in part of their training to learn what the 5-2-1-0 program is and what it stands for.” (C2)
- “…the health and wellness community has been integral in pushing the messaging out. They have adopted it amongst their own practices. Many of our practitioners, which is great in terms of sustainability. A number of them have taken it and embraced it within their own practices. Thinking of the [partner name] and [partner name]. Many of them have taken it under their own umbrellas, their own messaging, which is nice to see.” (D2)
- “I think with sustainability, what I think is some of the changes that [local coordinator name] has been mentioning with regards to the food and beverage policy. It sounds like the initial hurdle was a bit challenging, but once the initial changes have been implemented that is huge in making sure that the community is meeting the Live 5-2-1-0 around the recreation facilities.” (C2)

## 4. Discussion

Our study sought to evaluate the effect of a novel KE model (RE-FRAME) in scaling up the implementation of a community-based participatory childhood obesity prevention initiative and to describe the mechanisms by which the KE model increased community capacity. Our findings show that RE-FRAME supported scale-up of Live 5-2-1-0 in new partner communities via multi-sectoral partnership development that gave rise to community-led action to share and support Live 5-2-1-0. Further, RE-FRAME supported continued local action in existing communities, demonstrating its impact on sustainability [13]. Perceived community capacity increased over the duration of this study and emerged as a key component to initiating implementation in new communities and sustaining action in existing communities, particularly in the domains of leadership, community structure, and role of external support. Key to increasing community capacity were:

(i) a common approach and agenda (i.e., Live 5-2-1-0) that all sectors of a community could support and that facilitated cross-sectoral collaborative projects (i.e., playboxes).
(ii) community champions who sought and advocated for opportunities to integrate the initiative into the community’s make-up, sustaining momentum, building credibility and buy-in, and cultivating networks of stakeholder advocates.

These key factors fostered consistency of the Live 5-2-1-0 message and brand so that it was recognized across the community and became part of ‘what they do’. This entrenchment and integration of the message, as well as supportive action that achieved policy, programmatic or environmental change, were essential to sustainability of the initiative. Interestingly, new communities reported being ‘nearly there’, to ‘we’re there’ at baseline for community-capacity domains that directly aligned with elements of RE-FRAME (i.e., resources; skills, knowledge and learning; and links with others) confirming the importance of SCOPE’s support both at initiation and for maintenance of Live 5-2-1-0.

Community-based participatory approaches to childhood obesity prevention that are informed by the social-ecological model and that utilize a systems approach are becoming increasingly recognized as best practice for preventing childhood obesity [23,24,25,26]. There is a significant gap in the literature however on how to initiate, sustain, and scale-up a multi-sectoral community-led childhood obesity prevention effort. Our publications to date have described the use of Collective Impact to frame the implementation of Live 5-2-1-0 [12] as well as the creation of a novel KE model (RE-FRAME) that provided a scaffold upon which to describe our implementation processes [13]. This paper further adds to this growing literature by describing the role of and mechanisms by which community capacity building facilitates the initiation and maintenance of community-wide collective action to prevent childhood obesity, and the role of RE-FRAME in supporting implementation and scale-up of the initiative.

**Figure 3:**
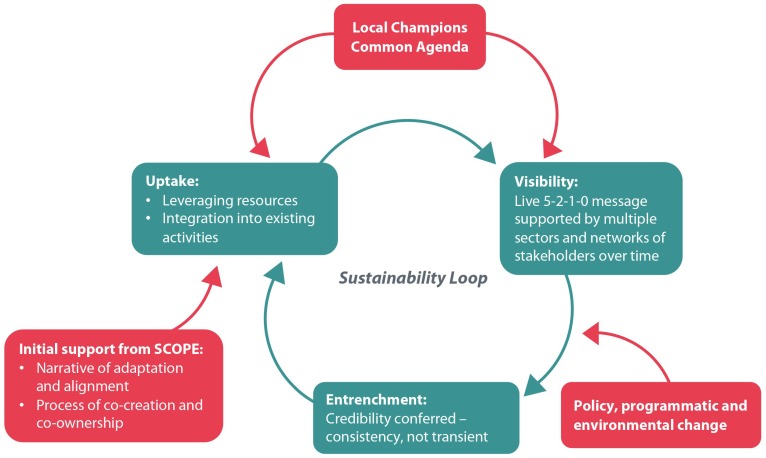
The reinforcing feedback loop that sustains Live 5-2-1-0 in a community.

From our data emerged a reinforcing feedback loop, with sustainability at its centre (Figure 3). The mechanisms by which community capacity was built (i.e., champion networks, co-creation and co-ownership, a common agenda/cause, and existing resources and ideas available through linkages with others and KE activities) led to uptake and buy-in of Live 5-2-1-0. This, in turn, enhanced the visibility of the initiative giving it more credibility, and when combined with the cross-sectoral consistency it offered, led to entrenchment of Live 5-2-1-0 into ‘what the community does’ via policy, programmatic or environmental change strategies, across multiple organizations and sectors.

Entrenchment leads to more uptake and buy-in by partners/champions, with a perpetual cycle of enhanced visibility and further entrenchment into the community’s make-up. The flexible and adaptable approach that SCOPE endorses in combination with the support offered by the SCOPE team to build community capacity using the RE-FRAME model and via its KE platform (resources, webinars, ad hoc coaching, etc.) jump starts this perpetual reinforcing cycle.

Importantly, the structure of the Live 5-2-1-0 initiative itself and the narrative received by communities surrounding ‘how it works’ are key elements for laying the groundwork of sustainability. The initiative is offered not as a predetermined and inflexible ‘program’ that a community must implement but cannot shape; rather, it offers an evidence-based, credible approach with associated resources that a community is welcome to take up if they align with a community-identified priority. This approach contains both a consistent, appealing, ready-made message as well as a shared ‘bank’ of resources, tools and ideas that communities are able to draw on. However, decisions about which resources or ideas to implement, how they should be adapted (or what unique new resources are needed), who should do the implementing, and how and when it will unfold remain the responsibility of the community. This empowers local champions to take a leadership role, and emphasizes the local investment in and ownership of resulting processes and projects.

This local ownership requires local capacity, and SCOPE supports the growth of this capacity through the tangible components that put the RE-FRAME model into practice. These include the direct ad-hoc coaching provided to community champions (including guidance on how to frame the narrative around Live 5-2-1-0 so that stakeholders understand its alignment and potential for integration with their existing work), the library of pre-existing Live 5-2-1-0 resources and tools, support for the co-creation of additional new and unique resources, the KE platform to connect communities to each other and leverage experiential learning, and expertise to assist with evaluation and progress tracking. This capacity-building approach is considered a best-practice principle for sustainability of obesity prevention initiatives [14]. Further, SCOPE’s role as the ‘backbone’ organization that facilitates capacity-building through KE with and between communities optimizes and enhances the collaborative interpersonal relationships that are foundational for effective uptake of knowledge [27].

In this way, SCOPE is also able to work in partnership with communities to ensure an effective yet feasible balance between ‘quality control’ and sustainable local ownership, even in the context of local staff turnover and dynamic stakeholder partnerships. As time has gone on, the breadth and refinement of community experience and available resources has grown; as a result, new communities are able to draw on this expanded support and ‘springboard’ into local engagement and action implementation more efficiently. This unique hybrid model of pre-established interventions/resources and new initiatives led by communities to meet their unique contexts is similar to the model described by Johnson-Shelton et al. in a community-school partnership project (CAST) to prevent childhood obesity [28]. The difference however in our work is that the pre-established research offered to communities interested in implementing Live 5-2-1-0 was co-created with our veteran communities, rather than being investigator-led. Nonetheless, Johnson-Shelton et al. report a similar experience to ours whereby the guidance, expertise and logistical support provided by their research-team ‘provided the project glue’ that held together shifting coalitions of community stakeholder partners and sustained the project’s direction and processes [28].

A key strength of our study is the mixed-methods approach and triangulation of quantitative and qualitative data that provided a comprehensive portrayal of communities’ experiences in implementing Live 5-2-1-0. Similarly, collection of qualitative data directly from community stakeholders/champions provided a more precise understanding of contextual factors that influence community capacity to implement community-wide interventions. Other strengths include the use of the validated Public Health Agency of Canada’s (PHAC)’s Community Capacity Building Tool (CCBT) and the novel Partnership Tracking Tool (PTT) that was created via in iterative process that integrated community stakeholder feedback to optimize the ease of data collection and ensure that insights generated from the data were meaningful to community stakeholders and leaders. Lastly, our intervention, the RE-FRAME model, was developed using the principles of community-based participatory research where community stakeholders participated in both its design and implementation.

This study is limited by the small sample size of communities which constrained our ability to do statistical testing on quantitative data. Similarly, local coordinators were limited to one per community however, our analysis elicited common themes across the eight interviews indicating that our data approached saturation. A key challenge in community-based research is staff turnover, and lack of time and resources for stakeholders to collect data. Even with the ease offered by the PTT, Community C was unable to provide complete data. Due to a hiatus in community stakeholder meetings, CCBT data at timepoint 2 for Community A were not available for analysis. Finally, the study periods were staggered for new and existing communities which may have impacted the comparability of findings between communities.

## 5. Conclusions

SCOPE, via the Live 5-2-1-0 initiative, aims to address the complex issue of childhood obesity via a multi-level intervention that applies the principles of CBPR and the framework of collective impact, while intersecting with systems thinking. Our work, in combination with our experiential learning on best practice, led us to develop an innovative KE model called RE-FRAME. Our research demonstrates that the components of RE-FRAME support knowledge exchange and translation that is integral to both initiating and sustaining a community-based childhood obesity prevention initiative. Further the outputs that result from the application of RE-FRAME (i.e., partnerships, organizational capacity, and adaptation) are cited in the literature as key to sustaining public health programs [29], and bolster our sustainability feedback loop (Figure 3). Ultimately, effective and efficient scale-up of Live 5-2-1-0 to more communities across BC will be informed by the learnings of this research. The study findings will guide the continued refinement of the model to best meet the community needs in confronting the dynamic and persistent challenge of childhood obesity.

The application of RE-FRAME however is not limited to childhood obesity prevention but rather has the potential to support a range of public health interventions aimed at solving complex issues. RE-FRAME shifts the focus of public health interventions towards community needs, community capacity building and empowerment, the mobilization of community champions, and co-creation and co-ownership so that the real-world contexts of a community can be addressed in intervention design, implementation and maintenance.

## Figures and Tables

**Table 1:** The RE-FRAME knowledge exchange model.

Model Component	Description
Reach	The extent of the key players, partnerships, and collaborations that are actively participating in the development and implementation of the project
Engagement	Developing, sustaining and fostering relationships that facilitate knowledge exchange and sharing
Facilitation, coaching, training	Technical support and sharing of expertise through active participation of knowledge-users and on-site coaching
Resources	Development of new or contextualization of existing resources to enhance self-efficacy and skills around administering childhood obesity prevention initiatives
Adaptation	Continuous tailoring and adapting of activities to local settings, contexts, needs and priorities
Mobilization of champions	Identifying and mobilizing key champions and early adopters who represent various community sectors
Exchange of knowledge	Multiple levels of continuous, bi-directional exchange of knowledge, learning, and expertise

**Table 2:** Domains assessed by the CCBT (PHAC).

CCBT Domain	Description	What It Looks Like
Participation	Active involvement of community stakeholders in improving health and well-being	Community members and stakeholders are involved in the initiative’s activities, such as making decisions and evaluation
Leadership	Engagement of, and support from, formal and informal local leaders	Effective leaders support and encourage community members’ involvement, share leadership, and foster networks that bring people with diverse skill sets together
Community Structures	Engagement of community groups and committees	Groups that foster belonging and give the community a chance to express views and exchange information
Role of External Supports	Support from local government, foundations, or regional health authorities	Supports can link communities with external resources (e.g., technical expertise) and build early community momentum
Asking Why	A process for uncovering root causes for community health issues and potential solutions	The community comes together to critically assess the social, political, and economic influences that result in differing health standards and conditions
Obtaining Resources	Finding time, money, leadership, volunteers, and information from both inside and outside the community	Accessing the internal and external resources needed for the initiative to succeed
Skills, Knowledge, Learning	Qualities in the project team and broader community stakeholders that are used and developed	Developing skills and knowledge and providing learning opportunities
Links with Others	Connections (links) among individuals and organizations through partnerships, networks, and coalitions	Networking, exchanging information, and taking joint action with actors across diverse sectors
Sense of Community	Trust, courage, and hope fostered by coming together to address shared community problems	Community members and stakeholders collaborating to develop, implement, and sustain solutions

Adapted by the authors from the PHAC CCBT User Manual [19].

**Table 3:** CCBT scores for Communities A–D.

	Mean Domain Score *							
Community	A		B		C		D	
Time Point	PRE	POST	PRE	POST	PRE	POST	PRE	POST
Participation	3.00	–	3.75	3.75	2.25	2.25	2.00	2.50
Leadership	3.33	–	2.00	4.00	1.33	2.00	2.33	2.67
Community Structures	2.33	–	3.00	4.00	1.67	2.67	1.67	2.33
Role of External Supports	3.33	–	3.33	4.00	2.75	4.00	3.25	3.75
Asking Why	2.67	–	4.00	4.00	2.33	1.67	1.33	3.00
Obtaining Resources	3.50	–	4.00	4.00	4.00	3.00	4.00	4.00
Skills, Knowledge, Learning	2.50	–	3.50	4.00	2.50	3.00	3.00	3.00
Links with Others	3.25	–	4.00	4.00	3.00	3.00	2.50	3.00
Sense of Community	3.00	–	4.00	4.00	2.00	3.00	3.00	3.00
**Overall**	2.99	–	3.51	3.97	2.43	2.73	2.56	3.03